# Systems biology coupled with label-free high-throughput detection as a novel approach for diagnosis of chronic obstructive pulmonary disease

**DOI:** 10.1186/1465-9921-10-29

**Published:** 2009-04-22

**Authors:** Joanna L Richens, Richard A Urbanowicz, Elizabeth AM Lunt, Rebecca Metcalf, Jonathan Corne, Lucy Fairclough, Paul O'Shea

**Affiliations:** 1 Cell Biophysics Group, School of Biology, The University of Nottingham, NG7 2RD, UK; 2 COPD Research Group, Institute of Infection, Immunity and Inflammation, The University of Nottingham, NG7 2UH, UK; 3 Department of Respiratory Medicine, Nottingham University Hospitals, Nottingham, UK

## Abstract

Chronic obstructive pulmonary disease (COPD) is a treatable and preventable disease state, characterised by progressive airflow limitation that is not fully reversible. Although COPD is primarily a disease of the lungs there is now an appreciation that many of the manifestations of disease are outside the lung, leading to the notion that COPD is a systemic disease. Currently, diagnosis of COPD relies on largely descriptive measures to enable classification, such as symptoms and lung function. Here the limitations of existing diagnostic strategies of COPD are discussed and systems biology approaches to diagnosis that build upon current molecular knowledge of the disease are described. These approaches rely on new 'label-free' sensing technologies, such as high-throughput surface plasmon resonance (SPR), that we also describe.

## Chronic Obstructive Pulmonary Disease

Chronic obstructive pulmonary disease (COPD) is a treatable and preventable condition characterised by progressive airflow limitation that is not fully reversible [1]. COPD is associated with an abnormal inflammatory response of the lungs to noxious particles or gases. This is primarily caused by tobacco smoking [2,3] but there is gathering evidence that additional factors predispose patients to COPD, such as genetic susceptibility, air pollution and other airborne irritants [4,5]. There may well be a genetic predisposition and also some food preservatives have also been implicated indicating that the underlying causality of the disease may not just reside in lung insult from the atmosphere [6]. COPD is projected to have a major effect on human health and worldwide by 2020 it is predicted to be the third most frequent cause of death [7].

COPD consists of three main respiratory pathologies; emphysema, respiratory bronchiolitis and chronic bronchitis. These separate and distinct pathologies illustrate the heterogeneity of COPD [8] and the importance of well defined COPD phenotypes [9]. Although COPD is primarily a disease of the lungs there is now an appreciation that many of the manifestations of disease are outside the lung, such as cachexia, skeletal muscle dysfunction, cardiovascular disease, depression and osteoporosis [10], leading to the concept that COPD is a systemic disease [11-15].

## Current Methods for Confirming a COPD Diagnosis

**Figure 1:**
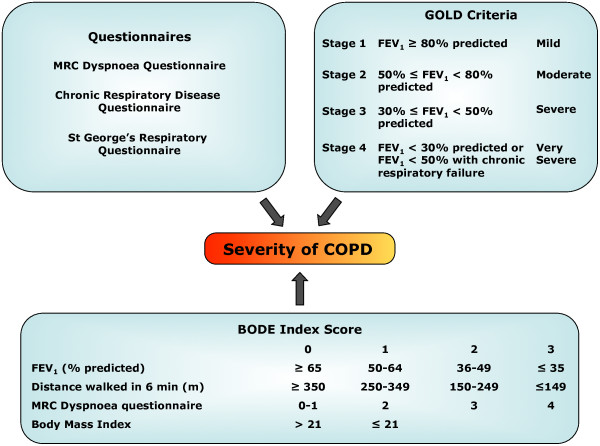
**The main methods currently used by clinicians to classify the severity of COPD**.

The diagnosis of COPD is based on the presence of typical symptoms of cough and shortness of breath, together with the presence of risk factors, and is confirmed by spirometry. A variety of methods (as outlined in Figure 1) are then used to classify the severity of disease, including questionnaires, GOLD and BODE Index.

The Global Initiative for Chronic Obstructive Lung Disease (GOLD) classifies COPD into four stages; mild, moderate, severe and very severe according to spirometric measurements [16]. Spirometry, however, is believed to correlate poorly with symptoms [17], quality of life [18], exacerbation frequency [19] and exercise intolerance [20].

A more recent and comprehensive method for assessing disease severity and prognosis of COPD is the BODE Index. This is a multidimensional grading system, which not only measures airflow obstruction (FEV_1_), but also incorporates body mass index (BMI), dyspnoea score and exercise capacity [21]. A comparison between the BODE and GOLD classifications shows that the BODE is a better predictor of hospitalisation [22] and death [21] than by GOLD.

There are conflicting views on the prevalence of COPD ranging from 3–12% [23] to 50% [24]. A major contributing factor to this may be that only one-third of physicians know the correct spirometric criteria according to GOLD [25] and only one-third of trained GPs and nurses trust their own spirometric interpretive skills [26]. Additionally, the technical limitations of the instruments used to undertake these spirometric measurements such as instrument variation and signal-to-noise ratio need to be considered [27,28]. Although spirometry is generally used to measure airflow obstruction, it has a number of limitations with regard to the detection and assessment of disease. Spirometry measures established airflow obstruction, which is likely to result from a long and continuous inflammatory process. Early use of therapeutic interventions, however, may be most helpful in attenuating the development of airway obstruction, which is not identifiable by spirometric tests. A single FEV_1 _measurement will give information on how much airway obstruction has already occurred, but will not give any information as to the current level of disease activity. At present, such information can only be obtained by serial measurements and assessment of the reduction in FEV_1 _over time. Finally, spirometry measures the end result of what may be a number of disease processes. It is known that patients vary considerably in their response to treatments, for example to inhaled corticosteroids [29], and it is possible that there are a number of pathways by which smoking and other exposures lead to the final state of COPD. An alternative diagnostic approach may help identify disease subtypes and allow for a more accurate distinction between COPD and chronic irreversible asthma [30].

## Biomarker Identification

In an effort to identify biomarkers of COPD, several groups have looked at genetic susceptibility (single nucleotide polymorphisms; SNPs), gene expression or protein expression. The observations from these studies have provided useful information and insights into the pathogenesis of COPD.

### Genetic susceptibility

As previously mentioned, COPD is associated with an abnormal inflammatory response of the lungs to noxious particles or gases. Due to the diverse response to these environmental insults, it is likely that genetic factors are important within the aetiology of COPD [31], but only severe alpha 1-antitrypsin deficiency is a proven genetic risk factor for COPD [32].

To date, studies have taken one of two approaches; they have either focused on candidate genes such as CCL5 [33] or taken a more holistic approach and completed genome-wide linkage analysis studies to identify regions of the genome that confer susceptibility [34]. The major considerations with any genetic study, however, are the large size required and the need for replication in a different, large data set. Using the focused approach Chappell *et al *have identified six haplotypes of the SERPINA1 gene that increases the risk of disease [35]. A recent genome-wide linkage analysis performed by Hersh *et al *identified a region on chromosome 1p that showed strong evidence of linkage to lung function traits [36]. Association analysis then identified TGFBR3 (betaglycan) as a potential susceptibility gene for COPD, which is supported by both murine and human microarray data.

### Gene Expression

Several researchers have examined gene expression profiles in an attempt to identify biomarkers, distinguish disease sub-types and generate candidates for further genetic and biological studies [37-45].

Spira *et al *reported genome-wide expression profiling of subjects with severe emphysema undergoing lung volume reduction surgery, which identified gene expression markers for severe emphysema as well as positive response to surgery [44]. Golpon *et al *used a similar approach and identified gene expression biomarkers distinguishing patients with α1-antitrypsin deficiency [41]. Pierrou and colleagues have identified a gene set of 200 transcripts dysregulated in COPD compared to healthy smokers [37]. As with most disease-focused microarray studies, however, there has been a lack of consistency in the identification of COPD gene expression biomarkers. For example, Egr-1 was identified in a microarray study as a gene over-expressed in emphysema subjects by Zhang *et al *[46]. Subsequently, Ning *et al*, using a combined microarray/SAGE approach, validated Egr-1 induction associated with COPD severity [40]. Ning *et al *went on to show that Egr-1 appears to contribute to disease pathogenesis, as it can regulate matrix-remodelling potential through fibroblast protease production. Bhattacharya *et al*, however, found no evidence of differential expression for Egr-1 in their population, although this study is one of the most promising to date, as the authors have presented the first gene expression biomarker for COPD validated in an independent data set [45]. This study, however, still has limitations, mainly due to the size of the sample population.

Overall, there is minimal overlap between differentially expressed genes among the different datasets. This problem highlights the complexity of expression profiling analysis in a human disease, such as COPD, with tissue heterogeneity and variable clinical phenotype. The non-overlapping gene datasets from these studies are due to several factors, including differences in sample acquisition, disease severity, sample size, tissue and cell components, and expression platforms [39].

### Protein Expression

Numerous groups have looked at protein expression, but most studies, due to technology limitations, have only analysed a limited set of proteins [47-52]. Shaker and colleagues examined six plasma proteins of known potential interest in COPD by enzyme-linked immunosorbant assay (ELISA) [48]. From this extremely selective reductionist approach they were able to show that some proteins were up-regulated and some were down-regulated, which emphasises the need for a more holistic approach to deliver a molecular fingerprint of disease. A larger scale analysis of proteins in COPD has been undertaken using two different techniques. Plymoth *et al*, by using a combination of replicate 2-dimensional gel separations, image annotation, and mass spectrometry identification, were able to investigate 406 proteins in bronchoalveolar lavage (BAL) that had the potential to identify smokers at risk of developing COPD [49]. These proteins showed expression patterns that were both up- and down-regulated. Pinto-Plata *et al *went a stage further and used serum on a 'Protein Microarray Platform' (PMP), which provided data on 143 serum proteins of potential interest [50]. This highlighted 24 proteins, which were up-regulated in disease, but it was acknowledged by the authors that the study was a proof of principle rather than a comprehensive analysis of all possible biomolecules related to COPD.

## Systems Biology: A New Approach to Disease Diagnosis and Management

Despite intensive research, definitive single disease-defining biomarkers for COPD remain elusive. Molecules shown to have a significant correlation with disease status often fail to accurately discriminate COPD from closely related diseases that display similar symptoms. As such, many of the potential biomarkers that have been suggested for COPD, including proteins [50,51,53], cytokines [48,50,54-65], antibodies [66], enzymes [50,67-69] and inhibitors [48], have also been implicated as potential targets in other lung diseases or general systemic inflammation [70-116] (Table 1). The difficulties encountered whilst searching for COPD biomarkers may be due in part to the complex nature of the disease, which comprises a broad spectrum of histopathological findings and respiratory symptoms [45]. Consequently, the probability of finding a single marker that is representative of all these processes is rather unlikely. Identification of single biomarkers is also hindered by the high level of variability in normal protein concentrations amongst individuals. This makes it difficult to establish the concentration of a single mediator that indicates disease onset [117,118]. Thus, it is essential to put isolated readings into context, i.e., does an elevated protein concentration indicate the presence of disease, or is it just a high but otherwise normal reading?

**Table 1:** Potential COPD biomarkers and other diseases in which they have been implicated.

**Potential COPD biomarker**	**Also implicated in**	**References**
Clara cell protein-10/16	Cystic fibrosis, general lung injury, lung cancer	[51,70,71]
C-Reactive Protein	Lung cancer, asthma	[50,72,73]
Endothelin-1	Asthma, idiopathic pulmonary fibrosis, lung cancer, heart disease	[53,74]
IFN-gamma	Pulmonary sarcoidosis, viral infections	[54,75,76]
IgG	Asthma, rheumatoid arthritis	[66,77,78]
IL-1	Rheumatoid arthritis, leukaemia	[55,79-81]
IL-4	Severe asthma	[56,82,83]
IL-6	Sarcoidosis, lung cancer	[57,84,85]
IL-8	Asthma, lung cancer, idiopathic interstitial pneumonia, sarcoidosis	[48,85-88]
IL-10	Burkitt lymphoma, asthma, sepsis	[58,89-91]
IL-12	Crohn's disease, systemic lupus erythematosus	[59,60,92,93]
IL-13	Asthma	[61,62,94,95]
IL-18	Asthma, sarcoidosis	[63,96,97]
IP-10	Sarcoidosis, asthma, SARS, tuberculous pleurisy	[64,98-101]
MMP-2	Lung cancer, asthma	[67,102-104]
MMP-12	Lung fibrosis, lung cancer	[68,105-107]
Myeloperoxidase	Lung cancer, cystic fibrosis	[69,108,109]
Neutrophil elastase	Systemic inflammatory response syndrome, lung cancer, cystic fibrosis	[50,110-112]
TIMP-1	Lung cancer, heart disease, asthma	[48,102,113,114]
TNF-alpha	Virus induced inflammation, HIV, asthma	[50,65,76,115,116]

The problems encountered with biomarker identification are not unique to COPD. Whilst the focus of biomarker studies over the last decade or so has primarily been placed on the use of individual molecular biomarkers as indicators of disease, this approach has only proved successful for a limited number of diseases including prostate and breast cancer where measurements of prostate specific antigen (PSA) and human epidermal growth factor receptor 2 (HER2) respectively are routinely used in diagnostic procedures [119,120]. New approaches to disease diagnosis in general, therefore, are required.

**Figure 2:**
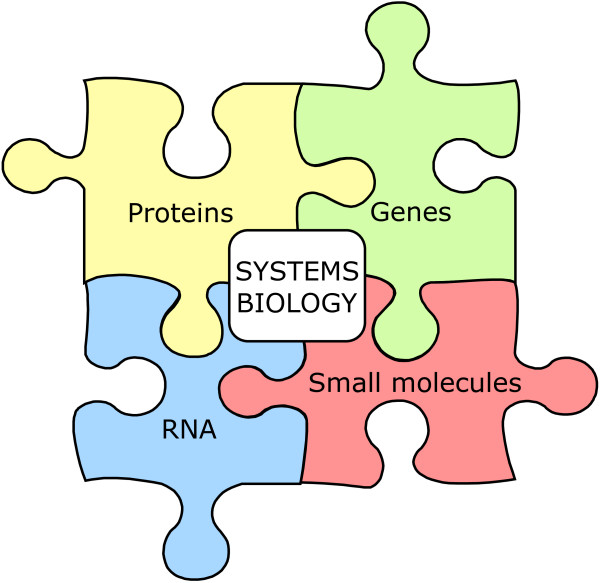
**Systems Biology: beginning to piece together the life sciences puzzle**.

Systems biology is a broad new paradigm that has recently entered the terminology of the life and biomedical sciences arena. It is an integrative approach focused on deciphering the relationship and the interactions between the gene, protein and cell elements of a biological system and how they impact on the function and behaviour of that system [121] (Figure 2). Traditional '-omics' fields, including genomics, proteomics, metabolomics and transcriptomics examine only one strand of the information available about an organism. Systems biology combines data from all these fields with bioinformatic, computational biology and engineering principles to examine organisms as systems of interconnecting networks. These networks will be modelled according to initial data obtained by traditional '-omics' and then revised through a combination of iterative refinement and bootstrapping (repeated random samples taken from a dataset) as described by Aderem [122] and Lucas [123]. By studying complex biological systems in this way, it is possible to identify emergent properties that are not demonstrated by individual '-omics' fields and cannot be predicted even with full understanding of the parts alone. A comprehensive understanding of these emergent properties requires systems-level perspectives not obtainable using simple reductionist approaches [122].

Studies have started to apply systems biology approaches and principles to decipher the pathways underlying complex diseases including Alzheimer's disease [124], polyarticular juvenile idiopathic arthritis [125], psychiatric disorders [126] and Sjögren's syndrome [127]. Application of the integrative approach provided by systems biology seems to offer a better route to understanding disease [128,129]. Currently, our understanding of systems biology is reaching a point whereby patterns of molecular behaviour are far clearer indicators of pathophysiological conditions than individual molecular markers [129]. Each disease possesses a unique molecular fingerprint that could be used diagnostically to differentiate it from diseases with closely related phenotypes. This novel concept, whilst still in its infancy, is being applied to cancer diagnosis [130] and is ideal for diagnosis of other complex diseases such as COPD.

Identification of a COPD-specific molecular fingerprint is a sizeable problem due to the heterogeneity of the disease and represents a huge undertaking. Different disease subtypes would each display slight, but measureable, variations of an overall COPD fingerprint. This fingerprint would also need to be sensitive enough to discriminate between COPD and other respiratory diseases e.g. chronic asthma, many of which display similar symptoms.

Initially, the COPD-specific molecular fingerprint would comprise biomolecules already associated with the disease, such as the RNA and protein molecules previously mentioned. Whilst these are the most well characterised disease targets, other molecular species may eventually form an integral part of a disease-specific molecular fingerprint. Targets such as SNPs [131], miRNA [132,133] and post-translational modifications [134,135] have all been shown to be important in disease pathology. Thus, a disease-specific molecular fingerprint would be a dynamic model that could be adapted to include such targets as new evidence becomes available of their involvement in COPD.

## Current Analytical Technologies

The feasibility of identifying disease-specific biomolecular patterns has been enhanced by the recent advent of proteomic and genomic technologies. Multi-parametric technologies, including bead-based assays (i.e., Luminex and Cytokine Bead Arrays), 2D gel electrophoresis, microarray platforms (both DNA and protein) and mass spectrometry, have provided the opportunities for a more holistic approach not previously possible using conventional technologies such as the enzyme-linked immunosorbent assay (ELISA) [136-140]. The implementation of these high-throughput technologies has vastly increased the prospects of biomarker research as they facilitate simultaneous analysis of multiple (often tens of thousands) potential biomarkers in minimal sample volumes with the potential for identifying novel targets not previously associated with the disease of interest. As such, they will be vital during the extremely complex task of identifying and revising disease-specific molecular fingerprints. Employment of systems biology approaches in routine diagnostic procedures, however, would require the availability of technologies that allow simultaneous detection of different molecular species e.g. both genes and proteins. The major disadvantage with the aforementioned techniques is the ability to detect only a single molecular species at once. Limitations with traditional proteomic and genomic technologies, particularly ELISA- and fluorescence-based systems, would be prohibitive to the production of systems that simultaneously detect multiple types of biomolecule. Such difficulties, including reagent limitations, the need for lengthy and complicated labeling, incubation and detection procedures and the potential for steric hindrance caused by the label at the binding site, could all be circumvented by the use of label-free technologies [141-143] such as surface plasmon resonance (SPR).

## Surface Plasmon Resonance (SPR)

### What is SPR?

**Figure 3:**
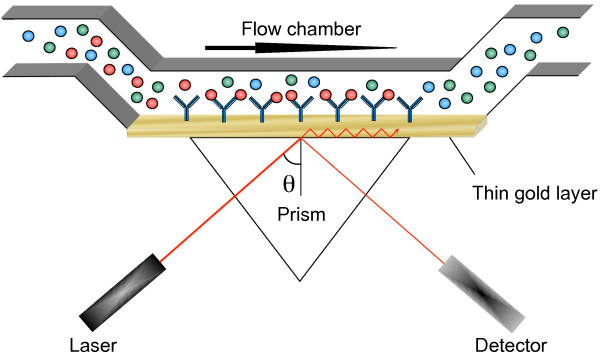
**Outline of a Surface Plasmon Resonance (SPR) system utilising a Kretschman-Raether configuration**. A system with this configuration facilitates label-free detection of biomolecules that bind in real-time. Biomolecules within the sample bind to ligands immobilised on the gold surface causing a change in the levels of the surface plasmon signals. Analysis of this change enables determination of both kinetic and analyte concentrations.

Surface plasmon resonance (SPR) polaritons are surface electromagnetic waves that propagate in a direction parallel to the interface between the metal surface and the external medium e.g., liquid. Since the wave exists on the boundary of the metal and the external liquid medium, these oscillations are very sensitive to any change of this boundary, such as the adsorption of molecules to the metal surface. This phenomenon enables the label-free, real-time detection of the interaction of biological molecules to the metal surface (usually gold) [144]. One frequently used configuration of the technology comprises a glass surface, coated with a thin gold film, which is attached to a prism (Figure 3). Chemical modification of the gold surface allows for the attachment of ligands for many different biomolecules [145-148]. Polarized light from a laser or other light source interacts with the gold surface at an angle greater than the critical angle (θ). Above this angle the light is coupled to electrons in the gold surface resulting in the propagation of surface plasmons along the surface. A surface plasmon only penetrates a short distance into the external medium (e.g., the aqueous environment in a flow cell) making it highly sensitive to changes on the surface of the gold but largely unaffected by processes in the bulk medium. Changes on the surface due to binding events can be readily monitored and have the potential to be used to measure concentrations, ligand-receptor binding affinities and association-dissociation kinetics of potentially thousands of proteins and genes rapidly and simultaneously [143].

### The use of SPR for the detection of biomolecules

The single great virtue of using SPR-based detection modalities is that they are label-free and thus do not require anything more for their identification apart from selective recognition on an appropriate chip surface. Coupling the appropriate surface chemistry for ligand attachment with SPR would allow detection of virtually any species of biomolecule. If the correct capture molecule is selected, SPR is specific enough to distinguish between different glycosylated forms of an antibody [149]. This flexibility, coupled with the potential for increased sensitivity [150], has led to an upsurge in the use of SPR technology. SPR has traditionally been used for identification of protein binding partners and characterisation of binding events [151-156]. It has been applied to the discovery and development of potential therapeutic agents [157-159] and characterisation of interactions between these compounds and their targets [160,161]. Additionally, it has been used to characterise the molecules, biochemical interactions and processes that may play a role in disease pathology [162-165].

More recently SPR has emerged as a powerful platform for biomarker studies and has been employed in the measurement of many biomolecules implicated in disease (Table 2). SPR detection systems have now been deployed in assays for a wide range of biomolecular species including proteins [166-172], antibodies [173], SNPs [174], sugars [175,176], narcotics [177,178], peptides [179,180], small molecules [181] and microRNAs [182]. These biomarkers have been identified within multiple types of clinical sample including mock samples [183], plasma [173,184-188], serum [189] and saliva [181,190]. Several of the studies mentioned in Table 2 have used SPR to detect biomarkers at clinically relevant concentrations highlighting the feasibility of using SPR in a clinical setting. For example, Nagel *et al *have been able to differentiate Lyme borreliosis infected patients from healthy donors by SPR analysis of Lyme borreliosis specific antibodies in blood serum samples [188]. Cho *et al *used SPR detection of CSFV antibodies to identify pigs infected with classical swine fever [191]. Vaisocherova *et al *devised an SPR assay for detection of the candidate pancreatic cancer marker activated cell leukocyte adhesion molecule (ALCAM) that can be used to distinguish between ALCAM levels in cancer and control sera [192]. The measurements made during the latter two studies were demonstrated to have comparative specificity and sensitivity to those undertaken with classical detection techniques [191,192]. SPR, however, has the additional benefits of being label-free, requiring no amplification step, having low sample requirement and high reusability, and requiring no sample pretreatment [192,193]. These advantages will in turn result in decreased experimental time, increased cost efficiency and simplification of detection protocols allowing lower user proficiency.

**Table 2:** Disease-specific biomarkers detectable by SPR

**Disease**	**Target molecule**	**Reference**
Cancer	Activated leukocyte cell adhesion molecule	[168]
	Ferritin	[193]
	Transgelin-2	[168]
	Cystatin C	[166]
Cardiovascular disease	B-type natriuretic peptide	[187]
	C-reactive protein	[169]
Cystic Fibrosis	W1282X mutation	[174]
Hepatocellular tumors	Alpha-fetoprotein	[185]
Inflammatory disease	Cystatin C	[166]
Lyme borreliosis	Pathogen specific antibodies	[188]
Myocardial infarction	Cardiac troponin I	[170,186,189]
	Myoglobin	[170,186]
Osteoporosis	N-telopeptide	[179,180]
Prostate cancer	Prostate specific antigen	[171,172]
Type 2 diabetes	Retinol binding protein 4	[184]
Viral meningitis	Beta2-microglobulin	[166]

## Systems Biology Approaches to COPD Diagnosis – Implementation of a working COPD specific microarray chip

**Figure 4:**
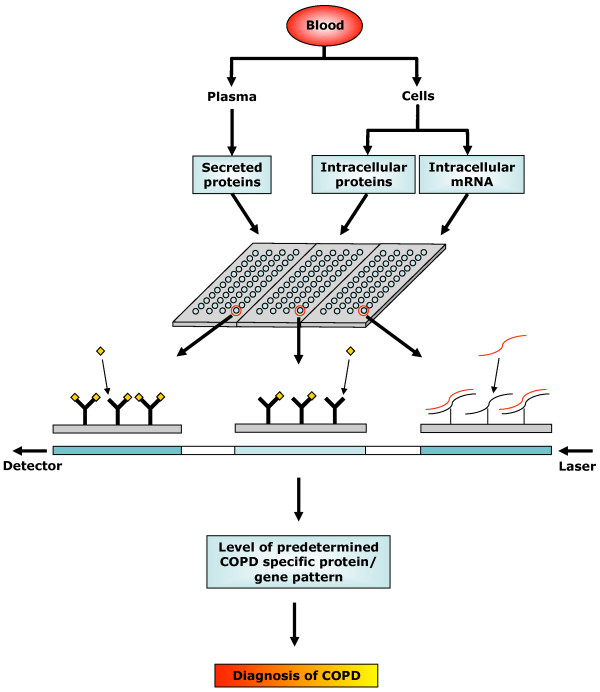
**A schematic representation demonstrating how a COPD-specific SPR microarray chip could be employed**. A small blood sample would be required, which would be separated into serum and cellular components using a microfluidic approach. Varying gene and protein expression would be monitored by changes in SPR enabling label free detection.

The principles of SPR, when combined with the use of an imaging step (SPR imaging; SPRi), allows a gold surface to be prepared in an array format providing the opportunity to study thousands of interactions rapidly and simultaneously [194]. SPRi could be employed in the development of a COPD specific microarray chip onto which ligands to the biomolecular components of the COPD-specific molecular fingerprint are arrayed (Figure 4). This diagnostic test examining levels of the biomolecules within the COPD molecular fingerprint would transform the accuracy, reliability and reproducibility of COPD diagnosis and assessment. We discuss below the broad methodology of the chip design and analytical implementation that offers much promise with disease detection and management.

### Target molecules

Initially the COPD specific microarray chip would be arrayed with antibodies, oligonucleotides and antigens as there is evidence of their ligands (proteins, RNA and antibodies respectively) being dysregulated in COPD [55,195-197]. Whilst the level of complexity of a biological system is vast, incorporating multiple cellular, genetic and molecular components, current approaches to disease-specific pattern analysis focus on deciphering panels of only one molecular component i.e., protein or mRNA [50,198,199]. For a more comprehensive depiction of the disease state, however, simultaneous examination of both the mRNA and protein levels of a molecule is vital as evidence suggests that correlation between the two can be poor [200,201]. In a study examining mRNA and protein expression in lung adenocarcinomas, only 21.4% of genes showed significant correlation with their corresponding protein [201]. Thus, both the mRNA and protein species of a molecule will be examined even if only one of these has been associated with disease. As the molecular fingerprint of COPD is further refined, the repertoire of detection would be adapted to allow for detection of single nucleotide polymorphisms (SNPs), microRNAs, peptides, enzymes/substrate interactions, small molecules (e.g. serotonin, vitamins, histamine), sugars or cell surface markers as appropriate.

### Clinical sample type

Another important factor to consider is the source of clinical sample being examined. Samples traditionally examined in cases of respiratory disease include induced sputum, BAL, lung tissue and, more recently, exhaled breath condensate (EBC). All of these sample types could potentially be analysed for patterns of biomarkers, but they are hindered by their invasiveness, cost or high level of variability [202]. The systemic manifestations of many complex diseases, including COPD [11,12], make analysis of body fluids an appealing option. In particular, the dynamic nature of blood means that it reflects the diverse physiological or pathological states of an individual. Coupled with its comparative ease of sampling, this makes the analysis of blood components the ultimate target for biomarker applications. Utilising blood samples would provide the opportunity to examine a full spectrum of molecular and cellular components within the disease-specific fingerprint including (but not exclusively) soluble proteins [50], cell types [203], cellular proteins/markers [204], autoantibodies [205], post-translational modifications [206] and circulating nucleic acids [207,208]. The proposed use of whole blood as a sample would require steps for separation on the basis of size and the ability to lyse cells to extract intracellular components. This could be achieved by coupling a microfluidic system, such as that previously described [209], to the chip to allow *in-situ *separation of the blood sample into plasma and cellular components.

Despite the huge potential of blood samples in diagnostic tests, some major challenges with its implementation need to be overcome. Past investigations into plasma biomarkers have been hindered by the fact that the plasma proteome is dominated by several highly abundant proteins, which mask proteins of much lower abundance identified as contributing to disease states [210-212]. This is not a trivial problem even in cases in which highly selective molecular-recognition-based protein identification technologies, such as those which are antibody-based, are employed. It is also important to consider other factors that may affect serum protein levels including psychological stress, time of blood sample collection, time since last meal, or uncontrolled differences in specimen handling [213,214]. Many of these limitations are beginning to be addressed [215,216] increasing the feasibility of comprehensive diagnostic testing in plasma. To this end, preliminary studies examining patterns of biomolecules, including proteins and autoantibodies, have been undertaken with some success for diseases such as graft versus host disease [217], chronic pancreatitis [198], brain cancer [218], lung cancer [219,220] and idiopathic pulmonary fibrosis (IPF) [221].

**Figure 5:**
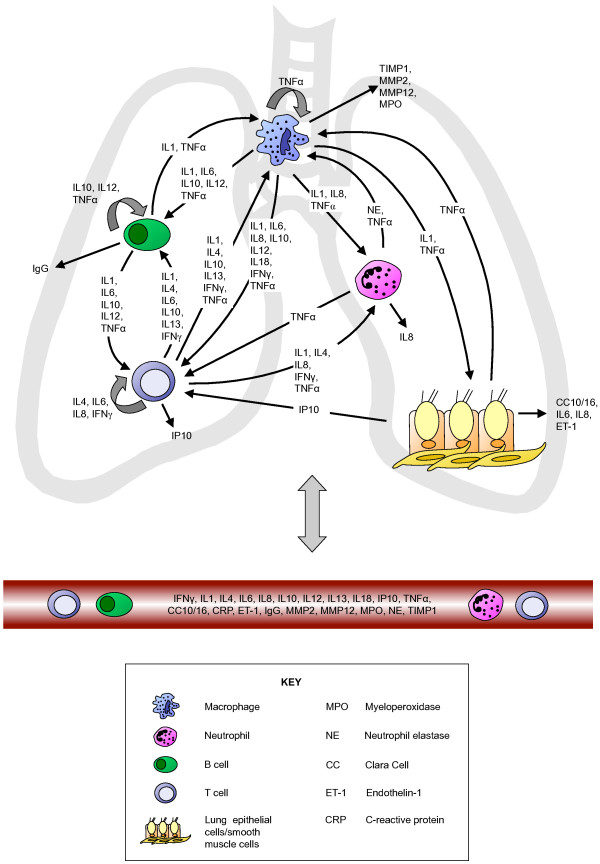
**Schematic representation of key molecules associated with COPD in the lung and periphery**. Analysis of these molecules at both the protein and gene level would form the basis of a molecular fingerprint of COPD for use in disease diagnosis.

With regards to COPD, there is preliminary evidence that patterns of biomarkers in the peripheral compartment could be used to distinguish patients with COPD. Increased concentrations of TNF-α and IL-6 have been demonstrated in the serum of stable COPD patients [222]. Pinto-Plata *et al *used a protein microarray platform to identify 24 serum proteins that were up-regulated in COPD [50] whilst Shaker *et al *demonstrated that down regulation, as well as up-regulation, of plasma proteins was indicative of COPD [48]. Man *et al *took this one step further and demonstrated that ratios of blood biomarkers, in this case fibronectin and CRP, are significantly associated with all-cause mortality of COPD patients [52]. Whilst such studies should be considered a proof of principle rather than a comprehensive analysis of all possible biomolecules related to COPD, this data provides evidence that a systems biology approach to COPD diagnosis and evaluation is attainable within blood. Additionally, whilst forming a complex network of interaction in the lung, all the potential COPD biomarkers identified in Table 1 have been detected within blood (Figure 5), although this has not always been in the context of COPD. These molecules, combined with those identified by the aforementioned studies, could provide the basis of a prototype peripheral compartment COPD molecular fingerprint.

### Defining, revising and analysing a molecular fingerprint

In addition to developing hardware with exquisite molecular sensitivity, the key to implementing advanced detection modalities is to include analytical protocols that are able to recognise complex biomolecular patterns made up of different molecular species and relate these to the disease condition under consideration e.g., COPD. Such analytical models now typically involve Bayesian inference approaches often starting with the hidden Markov model (HMM). This is essentially the simplest dynamic Bayesian network in which the system being studied is assumed to be a Markov process with unknown parameters. The challenge is to determine the hidden (i.e., disease) parameters from the observable molecular data so that the target condition of COPD can be identified. The Bayesian approach is particularly helpful with determination of the probability that any 'positive' result is actually a false positive. A systems biology approach to disease diagnosis strives to identify the presence of a molecular fingerprint of biomolecules that is not typically normal. Thus an observed biomolecular pattern from a suspected COPD patient is compared to a standardized 'healthy' pattern and diagnosed as having COPD or not. This approach is much more powerful than a diagnosis based on the presence of an altered concentration of a singular molecular marker e.g., PSA as it is less susceptible to the large variations in molecular marker concentration that naturally occur in any given population. The holistic measurement of a biomolecular pattern is more likely to reflect a disease condition than an individual molecular marker and, therefore, would augment the detection process. We are not alone in this vision, as others have also adopted this strategy as a way forward in molecular analysis. Alagaratnam *et al *are utilising Bayesian approaches to pursue muscular dystrophy diagnosis [223]. Similarly, the example we use above regarding PSA is also addressed using a systems analysis based on pattern-matching algorithms by groups in the US [224]. The problems with all these approaches however, are that they mostly rely on mass spectrometry for the molecular measurement and as such are expensive, require a significant investment in operator-skill and are less high-throughput than the SPR methodology we describe above. The latter point is extremely important if community screening is to be employed. Similarly, Bayesian approaches are not the only ways forward in mining the profile information. Other groups have discussed these approaches so we do not cover this in this review [225-227], but emphasise that it is the patterns of data that are important and not individual measurements. These analytical approaches are not just exclusive to the biomedical sciences as pattern analysis is central to much image analysis and recognition, such areas could well offer rich sources of analytical protocols.

## Potential Benefits

Adopting an SPR-based systems biology approach to COPD diagnosis would provide several distinct benefits. The potential for vastly improved disease diagnosis and classification is evident. As described earlier, whilst the current method of COPD diagnosis, i.e. spirometry, provides an indication of airway obstruction, it is insufficient for accurate disease evaluation, classification and subtyping. Analysis of biomolecular patterns would provide details on the molecular and cellular basis underlying the onset of COPD in an individual facilitating highly accurate disease diagnosis and classification. It would also provide a means by which the health of a COPD patient could be efficiently monitored. Inclusion of multiple molecular species within the molecular fingerprint will provide far more information than that obtained by analysis of a single molecular species. Highlighting the stage at which expression levels of a molecule vary would provide a greater insight into the causes of disease onset, identify important pathways for further examination and help direct future treatment strategies. Having a greater understanding of the molecular profiles underlying COPD would pave the way for personalized medicine where drug treatments are tailored towards the causal factors of disease for each individual.

Early symptoms of COPD are chronic cough and sputum production, which are often ignored by the patients and physicians, as they are thought to be a normal consequence of smoking [228]. It is not until an individual experiences further airway obstruction that spirometric testing will be undertaken, by which time irreversible damage will have occurred. The longer such symptoms are ignored, the worse the decline in lung function will be. With early detection, however, it may be possible to slow the age-related decline in lung function [229]. Thus, it is necessary to find ways in which to diagnose COPD when it is at a stage that is treatable and when smoking cessation will have an effect on prognosis. An SPR-based systems biology approach to COPD diagnosis would allow regular examination of biomolecular patterns in individuals with a family history of disease or those who are exposed to disease risk factors. Monitoring such individuals should facilitate significant improvements in early disease detection allowing enhanced drug intervention and anti-smoking measures at a time when treatment will be more effective, improving prospects for life expectancy and quality.

Finally, the benefits of biomolecular patterns would be seen in the field of drug discovery and development. Adoption of this strategy could be used to circumvent some of the problems associated with phase III clinical trials during drug development. Currently the assessment of therapeutic efficacy in phase III COPD drug trials involves following a large number of patients, over a long period of time, in order to measure decline in FEV_1_. The finding of a disease specific profile that accurately reflects current disease activity would reduce the need for such long-term, expensive, clinical trials [230] by allowing assessment of the immediate impact of potential drug therapeutics on disease mechanisms prior to an improvement of outwardly detectable symptoms. Improved understanding of the cellular and molecular basis of COPD pathogenesis would also potentially provide new therapeutic targets.

## Conclusion

Current methods for diagnosing COPD rely on spirometry combined with the use of questionnaires and other arbitrary measures for disease classification. Adopting a systems biology approach, whereby a disease defining molecular fingerprint is analysed, would increase the accuracy of disease diagnosis, aid earlier disease detection, allow for improved clarification of disease subtypes and allow automation for community screening.

## Competing interests

The authors declare that they have no competing interests.

## Authors' contributions

JLR and RAU mainly wrote the manuscript, as well as the revision, and contributed equally to the study. LF conceived of the review and wrote part of the manuscript. POS conceived of the review and edited and wrote part of the manuscript. EAML, JC and RM helped to draft the manuscript. All authors read and approved the final manuscript.
